# Gender Differences in Labour Losses Associated with Smoking-Related Mortality

**DOI:** 10.3390/ijerph16193644

**Published:** 2019-09-28

**Authors:** Juan Oliva-Moreno, Marta Trapero-Bertran, Luz María Peña-Longobardo

**Affiliations:** 1 Department of Economic Analysis. Faculty of Law and Social Sciences, Universidad de Castilla-La Mancha, 45071 Toledo, Spain; Juan.olivamoreno@uclm.es (J.O.-M.); luzmaria.pena@uclm.es (L.M.P.-L.); 2 Research Institute for Evaluation and Public Policies (IRAPP), Universitat Internacional de Catalunya, 08017 Barcelona, Spain

**Keywords:** labour productivity losses, smoking, tobacco, mortality, Spain, year of potential productive life lost, human capital, attributable fraction

## Abstract

The aim of this paper was to estimate the number of premature deaths, years of potential productive life lost (YPPLL) and labour losses attributable to tobacco smoking due to premature death by gender for the Spanish population. The human capital approach was applied. Employment, gross wage and death data were obtained from the Spanish National Institute of Statistics. Relative risks of death due to cigarette smoking and former smoking were applied. The base case used an annual discount rate of 3% and an annual labour productivity growth rate of 1%. Univariate deterministic sensitivity analysis was performed on discount rates and labour productivity growth rates. Between 2002 and 2016, smoking was estimated to cause around 13,171–13,781 annual deaths in the population under 65 years of age (legal retirement age) in Spain. This increase was mostly due to female deaths. YPPLLs for females have increased over the years, while for males they have fallen markedly. Labour losses associated with smoking mortality ranged from €2269 million in 2002 to €1541 in 2016 (base year 2016). In fact, labour productivity losses have decreased over the years for men (−39.8%) but increased sharply for women (101.6%). The evolution of monetary value of lost productivity due to smoking mortality shows clearly differentiated trends by gender.

## 1. Introduction

Tobacco use imposes a very large public health burden worldwide [1]. In the European Union, it is the largest avoidable health risk and the most significant cause of premature death, accounting for over 300,000 deaths per year [2]. On average, around half of smokers die 14 years earlier than non-smokers. Smoking is a major risk factor for at least two of the leading causes of mortality, cardiovascular diseases and cancer, and an important risk factor for many serious respiratory diseases [2].

However, patterns differ greatly depending on gender and age. According to World Bank data, between 2005 and 2016, smoking prevalence decreased by 32% for males, whereas for females it decreased by 11% [3]. According to the National Alcohol and Drugs Survey in Spain [4], in 2017 the highest prevalence for the previous 10 years was registered among men aged 15–34. In contrast, for women in this age group, the prevalence was the second lowest in the series. For individuals aged 35 to 64, the prevalence among men was the highest recorded since 2003, when 43.7% of men in this age group smoked tobacco daily. Regardless of sex and age, the survey showed an increase in the prevalence of daily tobacco use in 2017 compared with 2015. This increase is especially noticeable among men (increase of 4 points). Meanwhile, daily tobacco use among women aged 35 to 64 has shown a steady upward trend since 2013, reaching its highest point in the historical series in 2017 (30.5%). The percentage of women aged 35 to 64 who smoke daily exceeded the figure registered in the youngest group (15–34 years). Furthermore, a white paper addressing smoking from a gender perspective in Spain [5] had already stated that the epidemic patterns among men and women were different and that the burden of smoking-related diseases was more evident among men because women began to smoke several decades later. However, this situation is now changing and the differences need to be thoroughly studied.

Economic data and methods play an important role in tobacco control research, from estimating the costs of tobacco-related diseases and mortality to evaluating the cost-effectiveness of tobacco control interventions, programmes and policies. Some studies have quantified the economic cost that smoking imposes on society [6,7,8,9,10,11]. In fact, some of these papers have measured the economic impact of smoking reduction [11,12], or productivity losses caused by smoking [7,8,9,13] or both at the same time [6,10,14]. All papers concluded that investment in tobacco cessation interventions have led to reductions in smoking prevalence, savings in both healthcare and productivity costs as well as reductions in a high number of related diseases in the population. Thus, the amounts reported as saved by smoking reduction interventions account for billions of EUR or $US across countries.

Healthcare costs associated with diseases are a relevant indicator for measuring the impact of the tobacco epidemic. However, these costs are neither the only nor the most significant category of costs [7,8,9,13]. A report on damage caused by smoking in the EU27 found that the monetised cost of premature mortality, at standard value of statistical life (VSL) prices, amounted to 4.40% of total gross domestic product in the region. In fact, this was by far the largest component of the overall costs of smoking [15]. An alternative to the VSL approach to reveal the economic impact of smoking is the estimation of social costs associated with tobacco use [10,13]. One of their components is the output loss associated with dying prematurely [12]. While foregone economic output is only one of the dimensions of loss due to early death, it is by no means a negligible one. Indeed, lost economic output constitutes a considerable part of the economic burden of smoking, but many studies do not include it because there is no available information [16].

Smoking is also recognised as an important problem by the political establishment. Spain has been very active in the implementation of policies on tobacco use in public places. The Economic and Social Council of Spain, in its opinion on Act No. 28/2005, warned that public expenditure on tobacco-related costs was higher than tobacco excise duties [17]. In consequence, several laws and regulations have been passed over the last 10 years, most remarkably a total smoking ban in public places in 2010, with the aim of increasing the life expectancy and improving the quality of life of the non-smoking population. There are limited studies on morbidity and mortality and healthcare costs associated with smoking in Spain, although the scientific community acknowledge that it is the public health problem that causes the highest avoidable social costs [8,9]. However, these studies have concentrated mainly on calculating healthcare cost and on excess sick leave among smokers, but less on estimating economic losses associated with premature deaths related to tobacco use. Moreover, most studies have calculated the economic costs from a sample of workers, not at national level, and have not considered the possibility of age and gender differences.

Smoking, as a severe socioeconomic problem, not only results in an increase in healthcare costs, but also in costs due to productivity losses. There have been some studies that have calculated the number of deaths, years of potential life lost and productivity losses for the United States [18] and for Germany [12]. However, to our knowledge, there are no studies assessing the cost of foregone economic output due to premature mortality from smoking in the Spanish population and accounting for gender and age differences. The aim of this paper was to estimate the value of foregone economic output, number of deaths and potential years of working life lost attributable to premature deaths caused by tobacco use for the Spanish population, considering the evolution of tobacco use by gender. In order to achieve this objective, a microsimulation model of labour income, supported by the theoretical approach of human capital models [19], was developed. More precisely, the average earnings of a worker were used as a proxy for estimating labour productivity losses associated with leaving the labour market prematurely as a result of a health problem or a premature death. The hypothesis for testing this model was whether gender differences exist for the outcome measures calculated.

First, a section on methods details the human capital approach, data used and the simulation model built to calculate the main three outcome measures: number of premature deaths, years of potential productive life lost (YPPLL) and the loss of productivity attributable to tobacco use. The results section gives details on the results for each of these outcome measures. The discussion section compares the results obtained in this paper with other similar works already published. Lastly, a conclusions section provides a summary of the results obtained and suggestions for future research on this topic.

## 2. Materials and Methods 

### 2.1. Materials

Based on the availability of data in the various databases used in this analysis, the analysis has been performed for 2002, 2006, 2010 and 2016. This period was also chosen in order to include some years before and after the last economic crisis in Spain dated around 2008 and to include normative changes related to tobacco consumption. All calculations have been carried out for the total population and according to gender in order to test the hypothesis for gender differences across the outcome measure to be calculated.

For this purpose, we used data from the structural wage survey in Spain for 2002, 2006, 2010 and 2016 [20]. The concept of salary earnings includes both cash and in-kind payments made to workers for time worked or work done, along with payment for periods of time not worked, such as holidays and public holidays. This gross salary includes the pay-as-you-go social security contributions made by employers on behalf of the worker and withheld income tax. Furthermore, future flow of production lost was adjusted by employment rate (according to gender, age and year). These employment data were obtained from the Labour Force Survey of the National Statistics Institute (INE), where the employment rate is defined as the percentage of the employed population in relation to the working-age population [21]. The employed population were individuals of working age who had worked for at least one hour during the reference week in exchange for remuneration (salary, wage, business benefit, etc.) in cash or in kind. Individuals who have jobs but have been temporarily absent due to illness, holidays, etc. are also considered part of the employed population. The employment rate was adjusted by sex and age for each year considered. Data on mortality were obtained from the INE’s death statistics according to cause of death. Other data necessary for the calculation of this measure—i.e., number of deaths—were obtained from global burden of disease reports for 2005 to 2014. This source provides annual information on deaths by cause (International Classification of Diseases of the World Health Organization-10th revision) [22]. The diseases included in the analyses comprised malignant neoplasms, cardiovascular diseases and respiratory diseases, according to the evidence available [23,24]. Appendix A, Table A1, details the ICD-10 codes for the various diseases included [22].

### 2.2. Method

The human capital (HC) approach is employed for estimating labour losses. This is the method most commonly used in the vast majority of studies on the economic cost of smoking [11]. HC theory considers that the average wage obtained by the worker is a reasonable measure of (or the monetary value of) labour productivity [19]. Therefore, this method considers that the withdrawal of an individual’s labour due to premature death or permanent disability results in a loss to society of that individual’s future production. It is standard practice to estimate gross earnings, which includes payroll taxes and other employer-paid benefits—i.e., the full cost of employee compensation [25]. The theoretical justification for using total employer compensation per worker as a proxy for individual productivity is marginal productivity theory, according to which employers equate the marginal cost of employee time with the expected marginal contribution to output [25,26]. Additionally, although, ideally, such losses would be adjusted by as many variables as possible (e.g., sex, age, level of education, region, labour category, etc.), we were only able to obtain the cause of death by sex and age, as it was the case in the majority of studies applying this theory [27,28]. Therefore, labour productivity was approximated through remuneration in the labour market, using deaths and the employment rate for each year, by sex and age.

Taking into account the theory of human capital approach, a simulation model was constructed, once the age of death of each individual and the expected salaries were known, in order to calculate the present and future rate of production loss due to a premature death caused by tobacco. Firstly, years of potential life lost (YPLL) were estimated. Then, the number of deaths in each age group was multiplied by the average of the remaining life expectancy for each age group. Thus, the number of YPLL of the premature deaths of n individuals was calculated as follows:

$$YPLL^{j}=\sum_{i=1}^{n}d_{i}^{j}*L_{i}^{j}$$

where *j* is the gender, *d* is the deaths at the mid-point of each age group; and *L* is the average of remaining life expectancy for that age group.

Secondly, YPPLL were calculated. All deaths before 65 years of age (the legal age of retirement) were considered. Then, YPPLL was calculated by multiplying the number of deaths for a given age group by the expected remaining productive life years (until retirement age) for each age group. The number of YPPLL was calculated as follows:

$$YPPLL^{j}=\sum_{i=1}^{n}d_{i}^{j}*{\left(Wu-Wl\right)}_{i}^{j}$$

where *d* is the deaths at the mid-point of each age group; *Wu* is the upper limit of working age (65 years) and *Wl* is the age of death.

Finally, the calculated YPPLL was multiplied by sex and age-specific wages, adjusting by employment rate, between age of death and retirement age. Labour productivity losses (LP) can thus be estimated as follows:

$$LP^{j}=\sum_{i=1}^{n}YPPLL_{i}^{j}*S_{i}^{j}*e_{i}^{j}$$

where *S* is the wage adjusted by age and gender; and *e* is the employment rate adjusted by age and gender.

In order to conduct this analysis, the sample size was defined as the total number of deaths in the working age population by sex and cause of death for Spain, obtained from the National Statistics Institute [29]. To estimate premature deaths and YPPLL associated with tobacco consumption, we applied the attributable fraction (AF) method. The AF is the difference between overall average risk of the entire population (both exposed and unexposed) and average risk in the unexposed population, expressed as a fraction of the overall average risk. One of the most frequent interpretations of the AF is the proportion of disease risk or incidence (premature deaths in our study) that could be eliminated from the population if exposure (smoking) was eliminated. Attributable fractions were obtained from the Spanish Ministry of Health [30]. Total deaths for each underlying disease by sex and age were calculated. Deaths, YPPLL and labour productivity losses associated with tobacco use were only estimated in people over 35 years of age, as the AF was also only available for that age onward.

Therefore, for practical purposes, our analysis focused on labour productivity losses due to premature deaths attributable to tobacco use in the Spanish population aged 35 to 64 (the legal age of voluntary retirement is 65 years), by gender, in 2002, 2006, 2010 and 2016. In addition, 35 is the age threshold from which the effects of smoking on health was reflected in the data [30]. For that reason, our analysis focused on the population aged from 35 to 64.

For the baseline case, an annual discount rate of 3% and an annual labour productivity growth rate of 1% was applied to future income values obtained. A univariate deterministic sensitivity analysis was performed on two alternative discount rates (0% and 6%), as recommended in the Spanish Guidelines for Economic Evaluation [31,32] and two new labour productivity growth rates (0% and 2%). Finally, an additional sensitivity analysis was carried in order to isolate the epidemiological effect of the economic cycle so that we could test whether the labour productivity losses associated with tobacco use would be different if economic instability had not existed over the period considered. For this purpose, we re-estimated the losses with the average employment rate for the entire period instead of applying the employment rate for each year. Monetary values were converted to constant 2016 euros (base year) applying the GDP deflator.

## 3. Results

This section presents the estimations for number of premature deaths, YPPLL, and loss of productivity attributable to tobacco for the Spanish population over 35 years of age (), by gender, for the years 2002–2006–2010–2016. Some indicators to contextualize tobacco use in Spain are shown in Appendix A, Table A2.

### 3.1. Deaths in the Working Age Population

Table 1 shows the resulting estimations for the number of deaths associated with tobacco use in the working age population. In terms of weight, malignant neoplasms of the trachea, bronchus and lung are responsible for around 62% of all premature deaths in 2016, with respiratory diseases accounting for the smallest share of this estimation.

In terms of the evolution during the period considered, tobacco-related deaths from malignant neoplasms and respiratory diseases have increased by 14% and 8%, between 2002 and 2016, respectively, while deaths caused by cardiovascular diseases have decreased by 11%. Overall, the number of deaths attributable to tobacco increased by 4.6% between 2002 and 2016. However, for males the number of deaths decreased by 8.5% whereas for females, deaths increased by around 99%—i.e., they virtually doubled. Hence, there are gender differences in the number of deaths, and therefore the hypothesis established is confirmed. The explanation may lie in the increase in the number of cases of the diseases included in the model for women over the years and, as noted above, in the increase in smoking prevalence among women aged 35–64. The increase in deaths among women is mainly explained by an increase in the number of cancer deaths, which rose by 1326 from 2002 to 2016.

### 3.2. YPPLLs

Table 2 shows results in YPPLLs for the total population and by gender. In terms of weight, malignant neoplasms of the trachea, bronchus and lung are responsible for around 36% of all YPPLLs, with respiratory diseases accounting for the smallest share. Overall, the number of YPPLLs attributable to tobacco fell by 12.3% between 2002 and 2016. This reduction is mainly explained by the reduction in cardiovascular diseases, which accounted for almost 11,400 fewer YPPLLs. However, the trend by gender is markedly different. For males, the YPPLLs have decreased by 22%, while for females it has increased by 41.9%. Hence, there are gender differences in the number of YPPLLs, and the hypothesis established is therefore also confirmed for this outcome measure.

A comparison with the previous table reveals a downward trend in YPPLLs, while the trend of premature deaths has remained stable or increased slightly on average for males, but not for females, among whom YPPLLs have increased by almost 50%. This trend is explained by the fact that the average age of premature deaths in the working age population rose during the period. For instance, for the two main causes of death associated with smoking, lung cancer and acute myocardial infarction (AMI), the average age of death among men of working age increased from 55.5 years in 2002 to 57.4 years in 2016. Among women, the age rose from 51.4 to 55.9 years. In the case of the AMI, the average age of death went from 53.4 years in 2002 to 54.7 years in 2016 for men of working age and from 51.4 years to 54.0 years for women of working age. Hence, we are not seeing a decline in premature mortality; deaths are simply occurring at a later age.

### 3.3. Labour Losses

Table 3 shows the monetary values of estimated labour losses in constant 2016 euros. Annex B shows the same results but in nominal prices. The total labour productivity losses associated with tobacco amount to €2268.8 million, €1900.3 million, €1600.0 million and €1540.68 million for 2002, 2006, 2010 and 2016, respectively. Malignant neoplasms are the group of diseases that account for the highest proportion of labour loss associated with tobacco use, as they represent almost 55% of the total loss. Cardiovascular diseases (especially myocardial infarction and other ischaemic disease) are second, followed by respiratory diseases, accounting for 39% and 7% of the total productivity loss, respectively.

In general, the results indicate that labour losses associated with tobacco use have decreased from €2269 million to €1541 million during the period analysed, a reduction of 32%. Cardiovascular diseases account for the highest reduction in losses, as losses due to these diseases have decreased by €323 million, equivalent to more than 36%. Losses associated with cancers have also decreased by 30% over the last 14 years, dropping from €1229 million to €858 million.

Again, the results clearly differ by gender. Although the total reduction of labour losses is 32%, the decrease for males was almost 40%, but for females, the labour losses rose by more than 100% during the period. Nevertheless, total losses among men are still much higher than among women. The favourable trend in the case of men and the unfavourable trend in the case of women, however, makes the relative weight shift from 94.5% vs. 5.5% in 2002 to 83.8% vs. 16.2% in 2016. There are also gender differences for labour losses, and therefore the hypothesis established is also confirmed for this outcome measure.

Appendix A, Table A3, Table A4 and Table A5 show the results in current values for each year, without applying the deflator (A3 for the base case and both, A4 and A5, for the sensitivity analysis). Table A6 and Table A7 in the supplementary material show the results obtained from the sensitivity analysis, applying an annual discount rate of 6% and an annual labour productivity growth rate of 0% (Table A6), and an annual discount rate of 0% and annual labour productivity growth rate of 2% (Table A7). Both tables confirm the gender differences encountered so far.

Table A8, in the appendix, shows the results from the analysis isolating the epidemiological effect of the economic cycle, applying the average employment rate for the entire period. from the results, it is clear that, in general, if the economic cycle is isolated, for 2002 the labour productivity losses would be €2,245.66 million, 1% lower in comparison with the estimates for the base case for that year. Likewise, for 2016, the labour losses would be 0.04% lower if the economic cycle is isolated, while for 2006 and 2010 the losses would be slightly higher than in those years without isolating the economic situation (5% and 4%, for 2006 and 2010, respectively).

## 4. Discussion

This work covers a gap in information in Spain concerning the economic impact of smoking by gender, beyond its healthcare costs. The revelation of social costs associated with smoking does not replace epidemiological, clinical and healthcare cost information. On the contrary, it complements it and sheds light on an unknown dimension (hidden social costs) that enables a deeper understanding of the effects of smoking. This information may be a useful tool for public decision-makers in the planning of their preventive strategies and policies, and it might also be incorporated into economic evaluations of treatments to encourage smoking cessation.

This study confirms the hypothesis of gender differences in number of deaths, YYPLL and labour losses, as did previously published literature for Germany [14]. Other studies have calculated these outcome measures for the United States [18]; however, gender differences were not highlighted and evaluated specifically, as we did in this study. The two previous studies were published more than a decade ago, so new evidence, such as that produced in this paper, was needed. Other literature on economic costs and productivity losses have been found in the literature, but none of those studies calculated the impact of labour losses, using deaths averted and YPPLLs [6,7,8,9,10,11,12,13,14]. Our results show that men continue to account for the greatest burden of premature deaths, YPPLLs and labour losses associated with smoking. However, the trend observed among men is favourable, whereas the opposite is true of women. This inverse effect is in line with the prevalence trends for the two genders. As previously noted, the prevalence of smoking among women aged 35 to 64 years increased by almost 5% from 2002 to 2016, whereas for men in the same age group it decreased by 2%.

There are two additional factors that help to explain our results. The first is an increase in the average age of death among people of working age. The second factor is not so positive and shows a different trend for men and women over the period: the weight of female deaths within total smoking-related deaths in the working age population increased. The explanation may lie in the increase in the number of cases of the diseases included in the model for women over the years and, as noted above, in the increase in smoking prevalence among women aged 35–64. For example, in the case of lung cancer, the percentage of female deaths out of total deaths in 2002 was 10.4%. In 2016, it was 26.5%. In the case of the AMI, the proportion rose from 10.3% in 2002 to 14.2% in 2016. This increase in the proportion of female deaths reduces labour losses, since the employment rate and wages are lower in the case of women. The favourable trend in estimated labour losses is thus due to a positive factor (increase in the age of death) and to another factor related to the functioning of the Spanish labour market. Care should therefore be taken in interpreting the results.

The overall evolution of labour losses shows a clear downward trend, owing to the favourable trend for men, but not for women. Nevertheless, even though labour losses are declining, they still have a very significant impact from an economic point of view. The weight of labour productivity losses caused by premature deaths attributable to tobacco represented 0.24% of GDP in 2002, 0.18% in 2006, 0.15% in 2010 and 0.14% in 2016. Regarding total health spending (public and private), the weight of labour productivity losses caused by premature deaths attributable to tobacco is comparable to 2.2% of total health expenditure in 2006, 1.6% in 2010 and 1.5% in 2016 (data on total health spending not available for 2002).

Although we do not have information on labour losses caused by temporary or permanent absence due to illness, using the data on distribution of labour productivity losses estimated by Peña-Longobardo et al. (2016) [33] for major groups of diseases for Spain in 2009, a preliminary extrapolation of the impact of smoking on the loss of labour productivity can be made. The estimate indicates productivity losses (premature mortality, temporary and permanent absence due to illness) on the order of €4436.6 million in 2002, €4339.6 million in 2006, €3825.6 million in 2010 and €3834.5 million in 2016 (nominal values). This would mean figures equivalent to 0.59%, 0.43%, 0.35% and 0.34% of Spanish GDP in the years 2002, 2006, 2010 and 2016, respectively, and 5.35%, 3.82% and 3.77% of total health expenditure for the years 2006, 2010 and 2016, respectively.

Broadly, although the figures estimated showed that smoking can have hideous consequences for the economic situation of a country, few national studies have focused on the economic impact of smoking. Other economic analysis carried out in Spain recommended increasing the market price of cigarettes, as the price is much lower than the mortality cost per pack of cigarettes. More precisely, this study showed that the private mortality cost of smoking is €78 per pack of cigarettes for men and €54 per pack for women, with a statistical life value among Spanish smokers of €3.78 million [34]. For this reason, any interventions or programmes focused on reducing tobacco use should be considered in decision-making. Hormigo et al. analysed the efficiency (in terms of both costs and benefits) of a school-based smoking prevention program in Barcelona, considering as benefits both healthcare costs and avoided productivity losses (with a focus on higher productivity and years of working life gained). The healthcare benefits per averted smoker were €1997.57, but the indirect benefits per averted smoker were €23,258, of which 91% was due to avoided productivity losses (€21,260 per person). The authors concluded that the benefits of school-based tobacco prevention programmes, in terms of healthcare costs and productivity losses avoided, are far greater than the costs and therefore recommended that such programmes should be implemented [35].

Some limitations of this study should be noted. The AF for mortality was used as a proxy for smoking-attributable healthcare utilisation and expenditure, as no relative risk estimates for morbidity were available. Clearly, this assumption might lead to an overestimation or underestimation for morbidity AFs, depending on the disease. However, the estimate used for AF has been published by the Ministry of Health of Spain and should be based on good quality evidence and therefore be a good proxy for calculating national impacts [30]. The number of deaths was obtained from the Spanish National Institute of Statistics. There are other international public sources providing the number of deaths that could have facilitated future comparisons of the results with the results of other countries, such as the global burden of disease estimates. Different sources of information could lead to different results estimations. The authors of this paper believed that the national estimates were more reliable for making national estimations, but probably if future comparisons need to be made with other countries, other international data sources would be recommendable. Additional evidence on other smoking-related diseases could emerge, and other estimates would therefore need to be calculated to account for the new evidence. Age differences were also detected by this study, but for practical reasons-showing the results disaggregated by age would result in an unmanageable number of tables-we have not included the results in this paper. Indeed, differences observed across age ranges would not imply different policy decisions or actions.

## 5. Conclusions

As the average age of death caused by tobacco use is low (working age), smoking has a significant economic impact for society as a whole in terms of labour losses. However, this impact differs by gender (strongly concentrated in men, but the trend observed for men is favourable, whereas the opposite is identified for women). Although the anti-smoking laws previously approved [17] have helped to decrease the prevalence of male smokers, this has not occurred in the case of females, leading to a considerable increase in productivity losses associated with premature deaths. These results are aligned with previously published work for Germany [12]. These findings would imply that careful attention needs to be payed to reinforcing smoking cessation interventions among women in order to mitigate the effect that smoking seems to have had in recent years in this subgroup of the population. Primary healthcare centres should probably pay special attention to strengthening the brief advice intervention for smoking cessation in the female population. Stronger intervention measures against smoking should be taken without delay to reduce the health impact of smoking, especially in the female population. The findings of this study may help to create evidence to highlight the economic problems associated with smoking in Spain. This could encourage further stricter public policies, such as stiffer tax policy. Additionally, the results from this paper could be used in the future to study whether future public investment in smoking cessation policies will be offset by the savings produced in terms of the economic impact of tobacco on society.

Several future lines of research should be considered. Firstly, it might be advisable to extrapolate this analysis to other European countries in order to better calculate the societal costs of smoking in Europe and contribute to better estimations of the efficiency of smoking cessation interventions. In order to calculate these productivity losses and incorporate them in an economic evaluation, it is important to consider differences in impact by gender. It is not expected that these estimates will change recommendations in terms of the efficiency of the smoking cessation interventions, but they could reveal the hidden cost of smoking and potential social savings derived from primary prevention of smoking. And secondary, prevention of diseases associated with this behaviour could also help to reinforce health policy decisions to invest in public health interventions across European countries. Spain is a country where the economic impact of smoking has scarcely been quantified. Therefore, further research on economic impact, including healthcare costs and informal care costs, should be undertaken in order to complement this information. Gender differences in these different types of costs should also be explored.

Any future strategies or programmes should take into account the productivity losses associated with premature deaths (with special emphasis on female deaths) due to smoking, so that any health policy decisions can lead to an efficient allocation of resources, considering a wider perspective of the consequences of tobacco use.

## Figures and Tables

**Table 1:** Deaths in working age population (35 to 64 years).

Disease	2002			2006			2010			2016		
	Total	Male	Female	Total	Male	Female	Total	Male	Female	Total	Male	Female
Cancer	7437	6592	845	7427	6491	935	8471	6817	1654	8483	6312	2171
Malignant neoplasms of trachea, bronchus and lung	5074	4511	563	5068	4480	588	5495	4768	1177	5894	4331	1563
Other neoplasms (*)	2364	2081	282	2358	2011	347	2526	2049	477	2589	1981	609
Cardiovascular diseases	4670	4056	614	4697	4023	674	4100	3460	639	4151	3451	700
Ischaemic heart disease (MI+ and other ischaemic diseases)	2593	2323	270	2572	2269	303	2198	1924	274	2146	1842	304
Other heart diseases	882	740	141	936	783	153	917	752	165	1006	816	189
Stroke	770	619	151	763	600	163	623	473	150	615	471	144
Other cardiovascular diseases (atherosclerosis, aortic aneurysm, other arterial diseases and diabetes mellitus)	426	375	51	426	371	55	362	312	50	384	322	62
Respiratory diseases	1064	929	135	965	815	150	953	772	181	1147	844	303
Chronic obstructive pulmonary disease (bronchitis, emphysema and chronic obstruction of the airways)	630	581	49	527	476	50	569	486	83	598	449	149
Other respiratory diseases (pneumonia and influenza and tuberculosis)	433	348	85	438	339	99	384	286	98	549	395	154
Total	13,171	11,578	1593	13,088	11,319	1759	13,524	11,050	2474	13,781	10,607	3174
% of deaths by gender out of total	100%	87.9%	12.1%	100%	86.5%	13.4%	100%	81.7%	18.3%	100%	77%	23%
Variation in number of premature deaths 2002–2016	-	-	-	-	-	-	-	-	-	4.6%	−8.4%	99.2%

Source: own calculations. MI: Myocardial infarction. (*) Lip, pharynx and oral cavity, esophagus, stomach, pancreas, larynx, cervix, kidney and renal pelvis, bladder, liver, colon and rectum, acute myeloid leukemia.

**Table 2:** Years of potential productive life lost (YPPLL) in working age population (35 to 64 years).

Disease	2002			2006			2010			2016		
	Total	Male	Female	Total	Male	Female	Total	Male	Female	Total	Male	Female
Cancer	74,568	63,738	10,830	73,203	61,883	11,320	74,990	57,756	17,234	69,128	49,773	19,356
Malignant neoplasms of trachea, bronchus and lung	50,623	42,982	7641	50,272	42,517	7755	52,265	39,453	12,812	47,245	33,049	14,196
Other neoplasms (*)	23,945	20,756	3189	22,931	19,366	3565	22,725	18,304	4422	21,883	16,724	5159
Cardiovascular diseases	53,181	44,627	8555	51,628	42,853	8775	43,924	36,181	7744	41,786	34,172	7614
Ischaemic heart disease (MI+ and other ischaemic diseases)	29,802	26,217	3585	29,013	25,271	3742	24,136	20,960	3176	21,899	18,683	3217
Other heart diseases	10,525	8542	1983	10,470	8401	2069	10,166	8101	2066	10,692	8509	2183
Stroke	8641	6357	2283	8266	5970	2296	6528	4559	1969	5981	4383	1598
Other cardiovascular diseases (atherosclerosis, aortic aneurysm, other arterial diseases and diabetes mellitus)	4213	3510	703	3880	3211	669	3094	2560	534	3214	2597	617
Respiratory diseases	9354	7831	1523	8294	6657	1637	7969	6130	1839	9279	6573	2706
Chronic obstructive pulmonary disease (bronchitis, emphysema and chronic obstruction of the airways)	4351	3847	505	3541	3092	449	3829	3070	759	3838	2834	1004
Other respiratory diseases (pneumonia and influenza and tuberculosis)	5003	3984	1019	4754	3565	1188	4140	3060	1080	5441	3739	1702
Total	137,103	116,195	20,908	133,125	111,393	21,733	126,883	100,067	26,817	120,193	90,518	29,676
% of YPPLL by gender out of total	100%	84.8%	15.2%	100%	83.7%	16.3%	100%	78.9%	21.1%	100%	75.3%	24.7%
Variation in the number of YPPLL 2002–2016	-	-	-	-	-	-	-	-	-	−12.3%	−22.1%	41.9%

Source: own calculations. MI: Myocardial infarction. (*) Lip, pharynx and oral cavity, oesophagus, stomach, pancreas, larynx, cervix, kidney and renal pelvis, bladder, liver, colon and rectum, acute myeloid leukaemia.

**Table 3:** Labour losses (constant millions of 2016 euros) in working age population (35 to 64 years).

Disease	2002			2006			2010			2016		
	Total	Male	Female	Total	Male	Female	Total	Male	Female	Total	Male	Female
Cancer	1229	1166	63	1042	975	67	920	793	127	858	699	159
Malignant neoplasms of the trachea, bronchus and lung	830	785	45	717	670	47	634	538	96	577	460	117
Other neoplasms (*)	399	381	18	324	304	20	285	254	31	281	239	42
Cardiovascular diseases	891	839	52	745	691	54	583	523	60	567	499	68
Ischaemic heart disease (MI+ and other ischaemic diseases)	518	496	22	435	412	23	331	307	24	305	276	29
Other heart diseases	174	162	12	148	135	13	133	117	16	143	124	19
Stroke	132	118	14	109	95	14	79	64	15	76	62	14
Other cardiovascular diseases (atherosclerosis, aortic aneurysm, other arterial diseases and diabetes mellitus)	67	63	4	54	50	4	38	34	4	43	37	6
Respiratory diseases	149	140	9	113	104	9	97	84	13	115	92	23
Chronic obstructive pulmonary disease (bronchitis, emphysema and chronic obstruction of the airways)	70	67	3	49	47	2	45	40	5	47	39	8
Other respiratory diseases (pneumonia and influenza and tuberculosis)	79	73	6	64	57	7	52	44	8	54	53	1
Total	2269	2144	124	1901	1770	131	1600	1399	201	1541	1291	25
% of labour losses by gender out of total	100%	94.5%	5.5%	100%	93.1%	6.9%	100%	87.5%	12.5%	100%	83.8%	16.2%
Variation in labour losses 2002–2016	-	-	-	-	-	-	-	-	-	−32.1%	−39.8%	101.6%

Source: own calculations. MI: Myocardial Infarction. (*) Lip, pharynx and oral cavity, oesophagus, stomach, pancreas, larynx, cervix, kidney and renal pelvis, bladder, liver, colon and rectum, acute myeloid leukaemia.

## Appendix A

**Table A1:** Smoking-related diseases considered in the analysis.

General Category	Specific Category (I)	Specific Category (II)	Disease Code (ICD-10)
Tumours	Lung cancer	Lung cancer	C33–C34
	Other cancers	Lip, pharynx and oral cavity, oesophagus, stomach, pancreas, larynx, cervix, kidney and renal pelvis, bladder, liver, colon and rectum, acute myeloid leukaemia	C00–C14 C15 C16 C18 C19 C20 C22 C25 C32 C53 C64 C65 C67 C92
Cardiovascular diseases	Ischaemic heart disease	MI+ and other ischaemic diseases	I20–I25
	Other heart diseases	Rheumatic heart disease, cardiopulmonary diseases and other forms of heart disease	I00–I09 I26–I51
	Stroke		I60–I69
	Other cardiovascular diseases	Atherosclerosis, aortic aneurysm, other arterial diseases and diabetes mellitus	I70–I78
Respiratory diseases	Chronic obstructive pulmonary disease	Bronchitis, emphysema and chronic obstruction of the airways	J40–J44
	Other respiratory diseases	Pneumonia and influenza and tuberculosis	J09–J18 A15–A19

Source: WHO, 2004.

**Table A2:** General description of some indicators.

Disease	2002	2006	2010	2016
	Males			
Employment rate (35–64 years)	79.6%	81.1%	72,0%	72.9%
Average wages (euros per year)	22,335	22,720	25,479	25,924
Proportion of smokers in total population over 16 years of age ^a^	37.48%	35.27%	33.82%	28.24%
Proportion of premature deaths out of total premature deaths (all causes) ^a^	25.85%	24.79%	25.77%	26.73%
	**Females**			
Employment rate (35–64 years)	43.4%	52.1%	54.5%	59.4%
Average wages (euros per year)	15,848	16,499	19,735	20,131
Proportion of smokers in total population over 16 years of age ^b^	24.62%	23.85%	23.45	20.80%
Proportion of premature deaths above total premature deaths (all causes)	8.85%	9.26%	12.89%	16.47%

^a^ These data are aligned with the estimates of the Spanish Ministry of Health [Ministerio de Sanidad, Servicios Sociales e Igualdad. Muertes atribuibles al consumo de tabaco Tobacco en España, 2000-2014. Madrid: Ministerio de Sanidad, Servicios Sociales e Igualdad, 2016]. This report estimated that in that five-year period, deaths attributable to tobacco accounted for 25% of total deaths among persons aged 35 to 64 years and 12% among persons aged 65 and over. ^b^ Due to lack of information for some years, data are for 2003, 2006, 2009 and 2017.

**Table A3:** Labour losses (base case) (millions of euros).

Disease	2002	2006	2010	2016
Cancer	975.75	972.26	909.70	858.24
Malignant neoplasms of the trachea, bronchus and lung	659.02	669.47	627.65	577.68
ther neoplasms (*)	316.72	302.79	282.06	280.56
Cardiovascular diseases	706.83	696.26	576.67	567.35
Ischaemic heart disease (MI+ other ischaemic diseases)	410.76	405.96	328.31	304.93
Other heart diseases	137.69	137.89	131.67	143.81
Stroke	105.08	102.30	78.78	76.30
Other cardiovascular diseases (atherosclerosis, aortic aneurysm, other arterial diseases and diabetes mellitus)	53.30	50.11	37.91	42.31
Respiratory diseases	118.23	105.73	96.73	114.99
Chronic obstructive pulmonary disease (bronchitis, emphysema and chronic obstruction of the airways)	55.21	45.68	44.89	47.57
Other respiratory diseases (pneumonia and influenza and tuberculosis)	63.02	60.05	51.84	67.42
Total	1800.81	1774.25	1583.10	1540.58

Source: own calculations.

**Table A4:** Labour losses (discount rate 6%; annual production rate 0%) (millions of euros).

Disease	2002	2006	2010	2016
Cancer	79,769	79,584	75,507	72,048
Malignant neoplasms of the trachea, bronchus and lung	53,968	54,819	52,227	48,712
Other neoplasms (*)	25,801	24,765	23,280	23,336
Cardiovascular diseases	56,523	55,837	46,362	46,027
Ischaemic heart disease (MI+ and other ischaemic diseases)	32,834	32,486	26,396	24,756
Other heart diseases	10,893	11,011	10,452	11,531
Stroke	8450	8212	6380	6232
Other cardiovascular diseases (atherosclerosis, aortic aneurysm, other arterial diseases and diabetes mellitus)	4347	4129	3134	3508
Respiratory diseases	9712	8705	8000	9595
Chronic obstructive pulmonary disease (bronchitis, emphysema and chronic obstruction of the airways)	4757	3926	3848	4115
Other respiratory diseases (pneumonia and influenza and tuberculosis)	4955	4780	4152	5480
Total	146,004	144,127	129,868	127,670

Source: own calculations.

**Table A5:** Labour losses (discount rate 0%; annual production rate 2%) (millions of euros).

Disease	2002	2006	2010	2016
Cancer	124,761	124,108	113,817	106,029
Malignant neoplasms of the trachea, bronchus and lung	84,077	85,406	78,258	70,931
Other neoplasms (*)	40,683	38,702	35,559	35,098
Cardiovascular diseases	93,224	91,323	75,222	73,243
Ischemic heart disease (MI+ and other ischaemic diseases)	54,046	53,342	42,766	39,271
Other heart diseases	18,386	18,201	17,488	18,873
Stroke	13,941	13,422	10,185	9775
Other cardiovascular diseases (atherosclerosis, aortic aneurysm, other arterial diseases and diabetes mellitus)	6851	6358	4784	5324
Respiratory diseases	151.04	134.58	122.00	143.61
Chronic obstructive pulmonary disease (bronchitis, emphysema and chronic obstruction of the airways)	65.99	54.88	54.01	56.60
Other respiratory diseases (pneumonia and influenza and tuberculosis)	85.05	79.71	67.99	87.01
Total	1924.05	1901.88	1656.80	1585.35

Source: own calculations.

**Table A6:** Sensitivity analysis l. Labour losses (constant 2016 euros) (millions of euros). Annual discount rate 6%. Annual labour productivity growth rate 0%.

Diseases	2002			2006			2010			2016		
	Total	Male	Female	Total	Male	Female	Total	Male	Female	Total	Male	Female
Cancer	1005	955	50	853	799	54	763	660	103	720	588	132
Malignant neoplasms of the trachea, bronchus and lung	680	644	36	587	550	37	528	450	78	487	390	97
Other neoplasms(*)	325	310	15	265	249	16	235	210	25	234	199	35
Cardiovascular diseases	712	671	41	598	555	43	468	420	48	460	405	55
Ischaemic heart disease (MI+ and other ischaemic diseases)	413	396	17	348	330	18	267	247	20	247	224	23
Other heart diseases	137	128	9	118	108	10	106	93	13	116	100	16
Stroke	106	95	11	88	77	11	64	52	12	62	51	11
Other cardiovascular diseases (atherosclerosis, aortic aneurysm, other arterial diseases and diabetes mellitus)	54	51	3	44	41	3	31	28	3	35	31	4
Respiratory diseases	122	115	7	94	86	8	81	70	11	96	77	19
Chronic obstructive pulmonary disease (bronchitis, emphysema and chronic obstruction of the airways)	60	58	2	42	40	2	39	35	4	41	34	7
Other respiratory diseases (pneumonia and influenza and tuberculosis)	63	58	5	52	46	6	42	35	7	55	43	12
Total	1839	1740	99	1544	1440	104	1313	1,150	163	1277	1071	206
% of labour losses by gender out of total	100%	94.6%	5.4%	100%	93.3%	6.7%	100%	87.6%	12.4%	100%	83.9%	16.1%
Variation in labour losses 2002–2016	-	-	-	-	-	-	-	-	-	−30.6%	−38.4%	108.1%

Source: own calculations. (*) Lip, pharynx and oral cavity, esophagus, stomach, pancreas, larynx, cervix, kidney and renal pelvis, bladder, liver, colon and rectum, acute myeloid leukaemia.

**Table A7:** Sensitivity Analysis II. Labour losses (constant 2016 euros) (millions of euros). Annual discount rate 0%. Annual labour productivity growth rate 2%.

Diseases	2002			2006			2010			2016		
	Total	Male	Female	Total	Male	Female	Total	Male	Female	Total	Male	Female
Cancer	1572	1488	84	1329	1241	88	1150	988	162	1060	860	200
Malignant neoplasms of the trachea, bronchus and lung	1059	999	60	915	853	62	791	668	123	709	562	147
Other neoplasms(*)	513	489	24	414	388	26	359	320	39	351	298	53
Cardiovascular diseases	1174	1104	70	978	906	72	761	682	79	733	644	89
Ischaemic heart disease (MI+ and other ischaemic diseases)	681	652	29	571	541	30	432	400	32	393	355	38
Other heart diseases	232	216	16	195	178	17	176	155	21	189	163	26
Stroke	175	156	19	144	125	19	103	83	20	97	79	18
Other cardiovascular diseases (atherosclerosis, aortic aneurysm, other arterial diseases and diabetes mellitus)	87	81	6	68	63	5	48	43	5	53	46	7
Respiratory diseases	190	178	12	145	132	13	124	106	18	143	114	29
Chronic obstructive pulmonary disease (bronchitis, emphysema and chronic obstruction of the airways)	83	79	4	59	56	3	54	48	6	57	47	10
Other respiratory diseases (Pneumonia and influenza and tuberculosis)	107	99	8	85	76	9	69	58	11	86	67	19
Total	2935	2770	165	2452	2280	172	2034	1775	259	1936	1618	318
% of labour losses by gender out of total	100%	94.4%	5.6%	100%	93.0%	7.0%	100%	87.3%	1.7%	100%	83.6%	16.4%
Variation in labour losses 2002–2016	-	-	-	-	-	-	-	-	-	−34%	−41.6%	92.7%

Source: own calculations. (*) Lip, pharynx and oral cavity, esophagus, stomach, pancreas, larynx, cervix, kidney and renal pelvis, bladder, liver, colon and rectum, acute myeloid leukaemia.

**Table A8:** Sensitivity Analysis III. Labour losses (constant 2016 euros) (millions of euros).

Diseases	2002	2006	2010	2016
Cancer	1216.29	988.64	955.67	852.99
Malignant neoplasms of the trachea, bronchus and lung	822.31	680.61	658.45	571.57
Other neoplasms (*)	393.97	308.03	297.21	281.42
Cardiovascular	881.67	709.02	607.08	574.14
Ischaemic heart disease (MI+ and other ischaemic diseases)	510.06	412.58	346.37	310.22
Other heart diseases	172.14	140.64	138.48	145.16
Stroke	132.82	104.82	82.23	76.11
Other cardiovascular diseases (atherosclerosis, aortic aneurysm, other arterial diseases and diabetes mellitus)	66.66	50.98	40.00	42.65
Respiratory diseases	147.71	107.64	101.78	114.10
Chronic obstructive pulmonary disease (bronchitis, emphysema and chronic obstruction of the airways)	68.85	46.22	47.46	47.26
Other respiratory diseases (pneumonia and influenza and tuberculosis)	78.86	61.42	54.32	66.84
Total	2245.66	1805.30	1664.53	1541.23

Source: own calculations. (*) Lip, pharynx and oral cavity, esophagus, stomach, pancreas, larynx, cervix, kidney and renal pelvis, bladder, liver, colon and rectum, acute myeloid leukaemia.
